# The role of body composition, cardiometabolic parameters, and resting substrate oxidation in protecting against metabolic syndrome in adolescents with obesity

**DOI:** 10.3389/fnut.2025.1624696

**Published:** 2025-07-30

**Authors:** Mattia D’Alleva, Stefano Lazzer, Maria De Martino, Lara Mari, Enrico Rejc, Simone Zaccaron, Jacopo Stafuzza, Miriam Isola, Adele Bondesan, Diana Caroli, Francesca Frigerio, Laura Abbruzzese, Enrica Ventura, Alessandro Sartorio

**Affiliations:** ^1^Department of Medicine, University of Udine, Udine, Italy; ^2^School of Sport Science, University of Udine, Udine, Italy; ^3^Department of Neurosciences, Biomedicine and Movement Sciences, University of Verona, Verona, Italy; ^4^Experimental Laboratory for Auxo-Endocrinological Research, Istituto Auxologico Italiano, Istituto di Ricovero e Cura a Carattere Scientifico (IRCCS), Piancavallo, Italy; ^5^Division of Auxology, Istituto Auxologico Italiano, Istituto di Ricovero e Cura a Carattere Scientifico (IRCCS), Piancavallo, Italy; ^6^Division of Eating and Nutrition Disorders, Istituto Auxologico Italiano, Istituto di Ricovero e Cura a Carattere Scientifico (IRCCS), Piancavallo, Italy

**Keywords:** obesity, metabolic syndrome, respiratory quotient, cardiometabolic index, fat-free mass

## Abstract

**Purpose:**

The aetiology of metabolic syndrome (MetS) in young people involves a complex interplay between lifestyle, body composition, and cardiometabolic risk factors. The present study aimed to explore the relationships between anthropometric characteristics, body composition, cardiometabolic parameters and resting substrate metabolism in the development of MetS in severely adolescents with obesity.

**Methods:**

Seven hundred and thirty adolescents with obesity (mean age: 14.6 ± 2.1 years, BMI > 97th percentile for gender and age) were included in this study. Body composition analysis was obtained using tetrapolar bioelectrical impedance analysis (BIA), while resting substrate oxidation was measured using an indirect calorimeter.

**Results:**

MetS was present in 27% of the participants. Compared to those without MetS, adolescents with MetS had significantly higher body mass (+15 kg, *p* < 0.001), fat-free mass (FFM; +6 kg, *p* < 0.001), fat mass (+9 kg, *p* < 0.001), carbohydrate oxidation at rest (CHO; +0.02 g·min^−1^, *p* = 0.015), and Homeostasis Model Assessment for Insulin Resistance (HOMA-IR; +0.8, *p* < 0.001). In adjusted-univariate logistic regression, HOMA-IR (OR: 1.22; 95% CI: 1.12–1.34, *p* < 0.001) was associated with higher odds of MetS. Conversely, higher FFM percentage (OR: 0.96; 95% CI: 0.93–0.99, *p* = 0.003) and HDL cholesterol levels (OR: 0.83; 95% CI: 0.81–0.86, *p* = 0.003) were protective.

**Conclusion:**

In adolescents with severe obesity, resting carbohydrate oxidation and HOMA-IR emerged as independent risk factors for MetS, offering additional insight beyond conventional anthropometric and lipid indicators. Conversely, higher FFM and HDL cholesterol levels appeared to exert a protective effect. These findings underscore the importance of incorporating metabolic and body composition variables into MetS risk models and support the promotion of targeted interventions, such as endurance and resistance training, to address modifiable risk factors and reduce the likelihood of developing MetS.

## Introduction

Obesity is one of the major global health problems, affecting millions of individuals worldwide, with a particularly alarming prevalence among children and adolescents (1). Its primary causes include sedentary behavior, unhealthy dietary patterns, and insufficient physical activity (2). In paediatric populations, obesity is frequently associated with metabolic abnormalities such as insulin resistance, dyslipidaemia and hypertension, hallmark features of metabolic syndrome (MetS) (3). Cross-sectional studies have reported MetS prevalence rates ranging from 10 to 38% among children and adolescents with obesity (4, 5).

These metabolic disturbances are primarily driven by excessive fat mass (FM), particularly visceral adiposity (6). Visceral fat plays a key role in metabolic dysfunction through the secretion of adipokines involved in the pathogenesis of cardiometabolic diseases (7). Furthermore, increased FM promotes the accumulation of lipid intermediates, such as ceramides and diacylglycerols, within skeletal muscle (8). Combined with physical inactivity, this accumulation contributes to reduced mitochondrial density, enhanced adipogenesis, and decreased activity of key enzymes involved in aerobic energy production and fatty acid oxidation (9, 10). As a result, fat oxidation (FAT) at rest may be impaired (11, 12), and the ability to shift toward carbohydrate oxidation (CHO) in response to insulin stimulation is diminished (13). This impaired capacity to adjust substrate oxidation according to substrate availability, known as metabolic inflexibility, is typically characterised by an elevated respiratory exchange ratio (RER) at rest, reflecting a predominant reliance on carbohydrate metabolism (9). This phenomenon has been observed in individuals with metabolically unhealthy obesity (12). Indeed, resting substrate oxidation may provide additional predictive value for cardiometabolic risk, as it reflects both mitochondrial efficiency and the ability to utilise fat as an energy source in basal conditions (14). An elevated CHO oxidation rate at rest, may suggest impaired lipid oxidation and insulin resistance, both of which are implicated in the pathophysiology of MetS (15). Moreover, alterations in substrate utilisation may precede clinically evident metabolic dysfunction, offering an earlier window for risk identification (10). Nonetheless, findings remain inconsistent, particularly in paediatric populations with obesity, and this potentially valuable marker has been largely underexplored in paediatric risk models (16).

These metabolic alterations may also contribute to a reduction in fat-free mass (FFM) (16), leading to a lower basal metabolic rate (BMR), as FFM is the principal determinant of BMR (17). Over time, this unfavourable metabolic profile may increase susceptibility to the development of MetS (18). Despite these associations, to the best of our knowledge, no studies have specifically examined the relationship between resting substrate oxidation (i.e., CHO and FAT oxidation) and MetS risk in adolescents with obesity.

Given the increasing prevalence of MetS in this population, several indirect indexes have been proposed for its early identification in both clinical and epidemiological settings. Body mass index (BMI) remains the most used parameter (19); however, it does not differentiate between FM and FFM and provides no information on fat distribution (20). In paediatric populations, age- and sex-adjusted BMI z-scores are typically used, although their association with cardiometabolic risk is non-linear (21). Other indexes, such as waist circumference (WC), which indirectly reflect both the quantity and distribution of adipose tissue, have been used to assess body composition and cardio-metabolic risk factors (22). The waist-to-height ratio (WHR) has also emerged as a reliable screening tool to identify MetS in the paediatric population (23). More recently, the Visceral Adiposity Index (VAI), a sex-specific algorithm incorporating anthropometric measures (BMI and WC) and lipid profile parameters [triglycerides and high-density lipoprotein cholesterol (HDL-C)], has been proposed as a novel marker of cardiometabolic risk (24). VAI reflects visceral fat accumulation and dyslipidaemia, and has been linked to insulin resistance, impaired glucose regulation, and an increased cardiovascular risk. Its utility in identifying MetS has also been confirmed in paediatric populations (25). However, none of these indexes incorporates direct assessments of body composition, such as FM and FFM. Instead, they rely on indirect anthropometric parameters, such as BMI or WC. Moreover, to date, no studies have explored the potential role of resting substrate oxidation parameters in developing metabolic indexes for use in paediatric populations to facilitate the early identification of MetS.

Therefore, the aims of the present study were (i) to evaluate the differences in body composition, cardiometabolic parameters, and substrate oxidation at rest in a large cohort of adolescents with obesity, with and without MetS; (ii) to assess which of the parameters mentioned above act as protective or risk factors for the development of MetS; and (iii) to propose a novel MetS index that incorporates direct measures of body composition and substrate oxidation at rest.

## Materials and methods

### Study group

A retrospective cohort study was conducted on 733 adolescents (mean age: 14.8 ± 2.1 years; Tanner stage: 3.8 ± 1.4; height: 1.63 ± 0.10 m; body mass: 101.6 ± 22.7 kg; BMI: 37.9 ± 6.2 kg m^−2^) with severe obesity [BMI z-score > 2, based on the Italian reference growth charts for age and sex (26)]. All participants were admitted to the Division of Auxology at the Istituto Auxologico Italiano, IRCCS, Piancavallo-Verbania, for a 3-week multidisciplinary body weight reduction program. Inclusion criteria were: (i) age between 10 and 19 years; (ii) BMI SDS ≥ 2.0 according to sex- and age-specific Italian reference charts (27); (iii) essential obesity; (iv) abstinence from alcohol; (v) unbalanced type 2 diabetes mellitus. Exclusion criteria included: (i) genetic or syndromic obesity; (ii) any alcohol consumption; (iii) infection with the hepatitis B or C virus; (iv) type 1 diabetes mellitus; (v) obesity secondary to endocrine disorders (i.e., hypothyroidism, Cushing’s disease or syndrome). The study was conducted in accordance with the Declaration of Helsinki and was approved by the territorial Ethics Committee no. 5, Lombardy Region, Italy (approval number: 141/25; date of approval: March 25, 2025; internal order code: 01C515; acronym: OSSIGRASSIMET). At the hospital admission, informed assent/consent had been obtained from all participants and their parents.

### Measurements

#### Physical characteristics and body composition measurement

At hospital admission, each subject underwent a medical history review and physical examination. Body mass (BM) was measured to the nearest 0.1 kg using an electronic scale (Selus, Italy), with participants wearing only light underwear. Stature was measured to the nearest 0.5 cm using a standardised Harpenden stadiometer (Holtain Ltd., UK). Body mass index (BMI) was calculated as weight (kg) divided by the square of height (m) (26). Waist circumference (WC) was measured in a standing position, midway between the lowest rib and the top of the iliac crest, after a gentle expiration, using a non-elastic, flexible measuring tape (28). Hip circumference (HC) was assessed at the point of greatest posterior protuberance (28).

Body composition was evaluated using a multifrequency tetrapolar bioelectrical impedance analyser (BIA, Human-IM Scan, DS-Medigroup, Milan, Italy), delivering a current of 800 μA at a frequency of 50 kHz. To minimise measurement error, all procedures were standardised to ensure validity, reproducibility, and precision. Measurements were conducted according to the method described in Lukasky et al. (29), after a 20 min rest in the supine position, with arms and legs relaxed and not touching each other. FFM was estimated using a validated prediction equation (30), and FM was calculated as the difference between body mass and FFM. Although BIA is a practical and widely used method to estimate FFM and FM in adolescents, its precision is limited by several factors. A systematic review reported that test–retest measurement error in percentage body fat can be as high as 7.5–13.4% in youth, with poor agreement with criterion methods like dual-energy X-ray absorptiometry (DEXA) (31). Furthermore, accuracy diminishes in adolescents with higher degrees of obesity: correlations with DXA remain moderate or low, and the ability to track changes in FM and FFM is reduced in individuals with severe obesity (32). Hence, while BIA is acceptable for group-level assessments, caution is warranted when interpreting individual-level data.

#### Basal metabolic rate

Basal metabolic rate (BMR) was assessed following an overnight fast using an open-circuit, indirect computerised calorimetry system (Vmax 29, Sensor Medics, Yorba Linda, CA, United States) equipped with a rigid, transparent, and ventilated canopy to ensure minimal air leakage and stable measurement conditions. Before each measurement session, the calorimeter was calibrated according to the manufacturer’s instructions using standard gas mixtures (i.e., 16% oxygen and 5% carbon dioxide) to ensure accuracy and reproducibility of gas exchange measurements. Energy expenditure was calculated from oxygen consumption (V’O_2_) and carbon dioxide production (V’CO_2_) using the equation of Weir (33). Data acquisition was conducted under controlled environmental conditions (room temperature, 22 ± 1°C; humidity, 50–60%) to minimise variability.

The substrate oxidation rate at rest was determined from V’O_2_ and V’CO_2_ values using the following equations (34):


$$\begin{array}{l} \text{FATrest}\;(g\;\min^{-1})=1.67\times V^{’}O_{2}\;(L\;\min^{-1})-1.67\\ \times V^{’}{\mathrm{CO}}_{2}\;(L\;\min^{-1})-0.307\times \mathrm{Pox} \end{array}$$

$$\begin{array}{l} \text{CHOrest}\;(g\;\min^{-1})=4.55\times V^{’}{\mathrm{CO}}_{2}\;(L\;\min^{-1})-3.21\\ \times V^{’}O_{2}\;(L\;\min^{-1})-0.459\times \mathrm{Pox} \end{array}$$

where Pox is the protein oxidation rate. The protein oxidation rate was estimated by assuming that protein oxidation contributed approximately 12% of resting energy expenditure (34):


$$\begin{array}{l} \text{Protein oxidation rate}\;(g\;\min^{-1})\\ =\text{energy expenditure}\;(\mathrm{kJ}\;\min^{-1})\\ \times 0.12/16.74\;(\mathrm{kJ}\;g^{-1}) \end{array}$$

Missing or inconsistent data points from the calorimetry recordings, such as brief signal loss or artefacts, were identified by visual inspection of the raw data and the software’s quality control flags. These segments were excluded from the analysis. If data loss exceeded 5% of the total recording time, the measurement was repeated. No imputation methods were applied; all calculations were performed on validated continuous data segments.

#### Blood pressure measurements

Diastolic and systolic blood pressure (BP) were measured using a standard mercury sphygmomanometer to the nearest 2 mmHg after 5 min of rest. The average of three measurements taken on different days was used. Blood pressure was assessed according to the IDF criteria for paediatric age (35).

### Laboratory analyses

Baseline blood samples were collected via venipuncture after a 12 h overnight fast on the second day of hospitalisation. Fasting glucose, fasting insulin, total cholesterol, high-density lipoprotein cholesterol (HDL-C), low-density lipoprotein cholesterol (LDL-C), very low-density lipoprotein cholesterol (VLDL-C), triglycerides (TG), and C-reactive protein (C-RP) were measured using standard techniques.

The Homeostasis Model Assessment Index—Insulin Resistance (HOMA-IR) was calculated using the following formula (36):


$$\text{HOMA}-\mathrm{IR}:[\begin{array}{l} \text{Fasting glucose}\;(\text{mmol}\;L^{-1})\\ \times \text{fasting insulin}\;(\mathrm{mU}\;{\mathrm{mL}}^{-1}) \end{array}]/22.5$$

### MetS indexes

According to the International Diabetes Federation (IDF) criteria (37), the diagnosis of MetS was made when three or more of the following risk factors are present: a WC ≥ 80 cm, fasting glucose (FPG) ≥ 100 mg/dL (5.55 mmoL/L) or on drug treatment for elevated glucose, systolic blood pressure (SBP) ≥ 130 mmHg or diastolic blood pressure (DBP) ≥ 85 mmHg or on antihypertensive drug treatment in a patient with a history of hypertension, fasting triglycerides (TG) ≥ 150 mg/dL (1.7 mmoL/L) or on drug treatment for elevated TG, and HDL-C < 50 mg/dL (1.3 mmoL/L) or on drug treatment for reduced HDL-C.

In addition, the following indexes were calculated according to the following formulas (22, 38–42):


Waist−to−hipratio(WHR)=WC(cm)/HC(cm)

Waist−to−height ratio(WtHR)=WC(cm)/height(cm)

$$\begin{array}{l} \text{Body mass}\;\mathrm{fat}\;\text{index}\;(\text{BMFI})=\mathrm{BMI}\;(\mathrm{kg}\;m^{-2})\\ \times \mathrm{FM}\;(\%)\times \mathrm{WC}\;(m) \end{array}$$

$$\begin{array}{l} \text{Cardiometabolic Index}\;(\mathrm{CMI})=\text{WtHR}\\ \times \mathrm{TG}\;(\text{mmol}\;L^{-1})/\mathrm{HDL}-C\;(\text{mmol}\;L^{-1}) \end{array}$$

$$\begin{array}{l} \text{Visceral adiposity index}\;(\mathrm{VAI})=(\begin{array}{l} \mathrm{WC}\;(\mathrm{cm})/36.58+1.89\\ \times \mathrm{BMI}\;(\mathrm{kg}\;m^{-2}) \end{array})\\ \times (\begin{array}{l} \mathrm{TG}\;(\text{mmol}\;L^{-1})/0.81\times 1.52/\mathrm{HDL}\\ -C\;(\text{mmol}\;L^{-1}) \end{array})\;\\ \text{female};(\mathrm{WC}\;(\mathrm{cm})/39.68+1.88\times \mathrm{BMI}\;(\mathrm{kg}\;m^{-2}))\\ \times (\mathrm{TG}\;(\text{mmol}\;L^{-1})/1.03\times 1.31/\mathrm{HDL}-C\;(\text{mmol}\;L^{-1}))\;\text{male} \end{array}$$

$$\begin{array}{l} \text{Metabolic syndrome}\;z\_\text{score}\;(\text{MetS}\;z\_\text{score})\\ =-4.9310+0.2804\times \mathrm{BMI}\;z-\text{score}-0.0257\\ \times \mathrm{HDL}-C+0.0189\times \mathrm{SBP}+0.6240^{*}\;\ln\;(\mathrm{TG})\\ +0.0140\times \text{fasting glucose},\text{male};-4.3757\\ +0.4849^{*}\;\mathrm{BMI}\;Z-\text{score}=0.0176\times \mathrm{HDL}-C\\ +0.0257\times \mathrm{SBP}+0.3172\times \ln\;(\mathrm{TG})\\ +0.0083\times \text{fasting glucose},\text{female} \end{array}$$

### Statistical analyses

A Shapiro–Wilk test was used to assess the normality of each continuous variable. Normally distributed data were presented as mean ± standard deviation, while non-normally distributed data were reported as median and interquartile range (IQR: 25th–75th percentile). Adjusted odds ratios (ORs) were calculated using a logistic regression model to evaluate the association between the odds of having MetS and body composition, cardiometabolic parameters, resting substrate oxidation, and various indexes. Age and sex were included as covariates to control for potential confounding effects. An OR greater than 1 indicates an increased likelihood of metabolic syndrome, while an OR less than 1 suggests a protective association. 95% confidence intervals (CIs), *p*-values and Cohen’s d effect sizes were reported. A metabolic syndrome risk score was developed using the following appropriately scaled variables: sex, age, BMI, FFM (in kg), FM (in kg), WHR, indirect calorimetry, CHO (%), and FAT (%). Variables were selected to represent distinct physiological domains and to minimise redundancy among predictors. The dataset was randomly split into a training set (70%) and a testing set (30%). A LASSO logistic regression model with metabolic syndrome as the outcome variable was applied to the training set and iterated 100 times on bootstrapped samples. LASSO logistic regression is a regularisation method that applies an L1 penalty, and it was selected for its ability to perform variable selection and regularisation simultaneously. For each variable, the selection frequency and the mean LASSO coefficient were recorded. Variables with a selection frequency ≥ 60% were retained for score construction, based on recommendations from stability selection methods (43). The coefficients were then normalised to scale from −20 to 20. The risk score was calculated by multiplying each selected and scaled variable by its corresponding normalised mean coefficient. Youden’s index was applied to identify the optimal threshold for the risk score. At this cut-off, sensitivity, specificity, positive predictive value (PPV) and negative predictive value (NPV) were estimated with their 95% confidence intervals. Model performance was evaluated on the testing set by computing the area under the ROC curve (AUC) along with its 95% confidence interval.

## Results

### Physical characteristics of the study group

The descriptive characteristics of the study group are shown in Table 1. According to the IDF criteria, the presence of MetS was found in 202 patients (28%). Patients with MetS+ had significantly higher values of BMI z-score (+11%, *p* < 0.001), FFM (kg) (+11%, *p* < 0.001), FM (kg) (+18%, *p* < 0.001), WC (+10%, *p* < 0.001), HC (+4%, *p* < 0.001), TG (+44%, *p* < 0.001), Fasting insulin (+33%, *p* < 0.001), HOMA-IR (+32%, *p* < 0.001), SBP (+8%, *p* < 0.001), basal metabolic rate (+13%, *p* < 0.001) and CHOrest (+8%, *p* = 0.010) (Table 2). Additionally, HDL-C levels were significantly lower in the MetS+ group (−20%, *p* < 0.001) (Table 2). No significant differences were observed between the two groups in fasting glucose, total cholesterol and other resting substrate oxidation parameters (Table 2). Obese adolescents with MetS+ showed higher values of WHR (+5%, *p* < 0.001), WHtR BMFI (+19%, *p* < 0.001), VAI (+96%, *p* < 0.001), CMI (+95%, *p* < 0.001) and MetS z-score (+55%, *p* < 0.001) than obese adolescents without MetS (MetS−) (*p* < 0.001) (Table 2).

**Table 1:** Descriptive statistics for the whole study group.

Characteristics	*n* = 733^a^
Female [*n* (%)]	440 (60%)
Age (*n*)	14.91 (13.08, 16.41)
Height (m)	1.63 (1.56, 1.69)
Body weight (kg)	97 (86, 116)
BMI (kg m^−2^)	37 (33, 41)
BMI z-score	2.99 (2.60, 3.41)
FFM (kg)	46 (41, 52)
FFM (%)	47.3 (43.3, 51.4)
FM (kg)	51 (43, 62)
FM (%)	52.7 (48.6, 56.7)
Waist circumference (cm)	114 (104, 124)
Hip circumference (cm)	121 (113, 129)
WHR	0.95 (0.90, 1.01)
Fasting glucose (mg dL^−1^)	81 (77, 85)
Total cholesterol (mg dL^−1^)	161 (142, 182)
HDL-C (mg dL^−1^)	42 (36, 49)
LDL-C (mg dL^−1^)	101 (84, 120)
VLDL-C (mg dL^−1^)	18 (13, 23)
Triglycerides (mg dL^−1^)	89 (67, 116)
C-reactive protein (mg dL^−1^)	0.40 (0.20, 0.70)
Fasting insulin (mU L^−1^)	14 (9, 19)
HOMA-IR	2.72 (1.78, 3.95)
Systolic blood pressure (mmHg)	120 (120, 130)
Diastolic blood pressure (mmHg)	80 (70, 80)
RER	0.80 (0.75, 0.86)
CHO rest (%)	36.9 (19.2, 54.1)
FAT rest (%)	66.6 (49.3, 84.4)
Basal metabolic rate (kcal day^−1^)	1,883 (1,669, 2,144)
Metabolic syndrome (*n*)	202 (28%)
Basal metabolic rate (kcal kg FFM^−1^)	40 (37, 44)
CHOrest (g min^-1^)	0.12 (0.07, 0.17)
FATrest (g min^−1^)	0.09 (0.06, 0.12)
BMFI (kg m^−1^)	22 (17, 28)
VAI (cm^2^)	1.85 (1.27, 2.65)
WtHR	0.70 (0.65, 0.75)
CMI	1.48 (1.02, 2.16)
MetS_zscore	1.43 (1.04, 1.84)

a *n* (%); median (Q1, Q3).

BMI, body mass index; FFM, fat-free mass; FM, fat mass; WHR, waist-to-hip ratio; HDL-C, high-density lipoprotein cholesterol; LDL-C, low-density lipoprotein cholesterol; VLDL-C, very low-density lipoprotein cholesterol; HOMA-IR, Homeostasis Model Assessment Index-Insulin Resistance; RER, resting exchange ratio; CHOrest, carbohydrate oxidation at rest; FATrest, fat oxidation at rest; BMFI, body mass fat index; VAI, visceral adiposity index; WHtR, waist-to-height ratio; CMI, cardiometabolic index; MetS_zscore, metabolic syndrome z score.

**Table 2:** Descriptive statistics for all adolescents without metabolic syndrome (MetS−), and with metabolic syndrome (MetS+).

	MetS−	MetS+	*p*-value^b^
	(*n* = 531^a^)	(*n* = 202^a^)	
Female [*n* (%)]	339 (64%)	101 (50%)	0.001
Age (*n*)	14.58 (12.75, 16.00)	15.66 (14.00, 16.83)	<0.001
Height (m)	1.61 (1.55, 1.67)	1.67 (1.61, 1.73)	<0.001
Body weight (kg)	94 (84, 109)	112 (96, 129)	<0.001
BMI (kg m^−2^)	36 (33, 40)	39 (36, 43)	<0.001
BMI z-score	2.91 (2.54, 3.30)	3.24 (2.88, 3.54)	<0.001
FFM (kg)	45 (40, 50)	50 (44, 60)	<0.001
FFM (%)	47.5 (43.6, 51.6)	46.6 (42.4, 50.8)	0.035
FM (kg)	49 (42, 59)	58 (49, 69)	<0.001
FM (%)	52.5 (48.4, 56.4)	53.4 (49.2, 57.6)	0.035
Waist circumference (cm)	111 (103, 121)	122 (113, 132)	<0.001
Hip circumference (cm)	119 (112, 127)	124 (118, 132)	<0.001
WHR	0.94 (0.88, 0.99)	0.99 (0.93, 1.04)	<0.001
Fasting glucose (mg dL^−1^)	81 (77, 85)	81 (78, 86)	0.497
Total cholesterol (mg dL^−1^)	160 (142, 181)	164 (139, 185)	0.621
HDL-C (mg dL^−1^)	44 (40, 51)	35 (32, 38)	<0.001
LDL-C (mg dL^−1^)	99 (83, 119)	106 (85, 126)	0.024
VLDL-C (mg dL^−1^)	16 (13, 21)	24 (18, 31)	<0.001
Triglycerides (mg dL^−1^)	82 (64, 104)	118 (89, 156)	<0.001
C-reactive protein (mg dL^−1^)	0.40 (0.20, 0.70)	0.30 (0.20, 0.70)	0.672
Fasting insulin (mU L^−1^)	12 (8, 18)	16 (12, 22)	<0.001
HOMA-IR	2.44 (1.64, 3.72)	3.23 (2.38, 4.45)	<0.001
Systolic blood pressure (mmHg)	120 (120, 130)	130 (130, 140)	<0.001
Diastolic blood pressure (mmHg)	80 (70, 80)	80 (80, 90)	<0.001
RER	0.80 (0.75, 0.86)	0.80 (0.76, 0.85)	0.370
CHO rest (%)	36.9 (19.2, 54.1)	40.3 (22.8, 54.1)	0.444
FAT rest (%)	66.6 (49.3, 84.4)	66.6 (49.3, 84.4)	0.256
Basal metabolic rate (kcal day^−1^)	1,835 (1,635, 2,064)	2,065 (1,768, 2,330)	<0.001
Basal metabolic rate (kcal kg FFM^−1^)	41 (37, 45)	40 (36, 44)	0.111
CHOrest (g min^-1^)	0.12 (0.07, 0.16)	0.13 (0.08, 0.19)	0.010
FATrest (g min^−1^)	0.09 (0.06, 0.12)	0.10 (0.07, 0.13)	0.142
BMFI (kg m^−1^)	21 (17, 26)	25 (20, 31)	<0.001
VAI (cm^2^)	1.57 (1.18, 2.16)	3.08 (2.13, 4.20)	<0.001
WtHR	0.69 (0.64, 0.74)	0.73 (0.68, 0.78)	<0.001
CMI	1.29 (0.92, 1.78)	2.51 (1.82, 3.48)	<0.001
MetS_zscore	1.24 (0.89, 1.58)	1.92 (1.61, 2.23)	<0.001

a *n* (%); median (Q1, Q3).

b Pearson’s Chi-squared test; Wilcoxon rank sum test.

BMI, body mass index; FFM, fat-free mass; FM, fat mass; WHR, waist-to-hip ratio; HDL-C, high-density lipoprotein cholesterol; LDL-C, low-density lipoprotein cholesterol; VLDL-C, very low-density lipoprotein cholesterol; HOMA-IR, Homeostasis Model Assessment Index-Insulin Resistance; RER, resting exchange ratio; CHOrest, carbohydrate oxidation at rest; FATrest, fat oxidation at rest; BMFI, body mass fat index; VAI, visceral adiposity index; WHtR, waist-to-height ratio; CMI, cardiometabolic index; MetS_zscore, metabolic syndrome z score.

### Protective and risk factors for MetS

**Figure 1:**
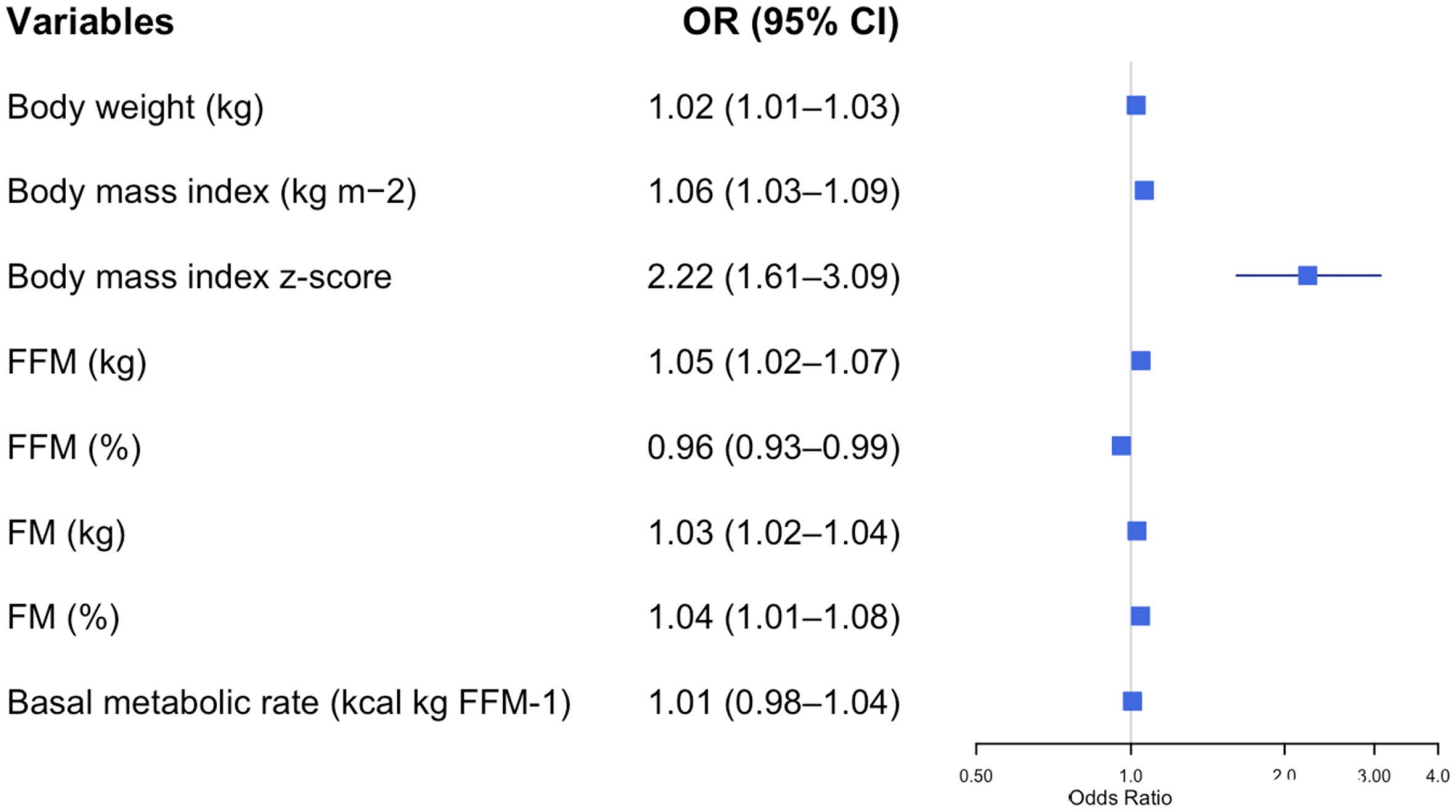
Forest plot of adjusted odds ratios (ORs) and 95% confidence intervals (CIs) for body composition parameters and metabolic syndrome risk in adolescents with obesity. Each row displays a specific index, with the square dot representing the adjusted-for-age odds ratio and the horizontal line extending from the dot indicating the 95% confidence interval. The plot includes a vertical reference line at an OR of 1.0, representing no effect. Predictors with confidence intervals that do not cross this line suggest a statistically significant association with obesity risk. The numerical values of the odds ratio and their CI are placed next to each index. FFM, free fat mass; FM, fat mass.

**Figure 2:**
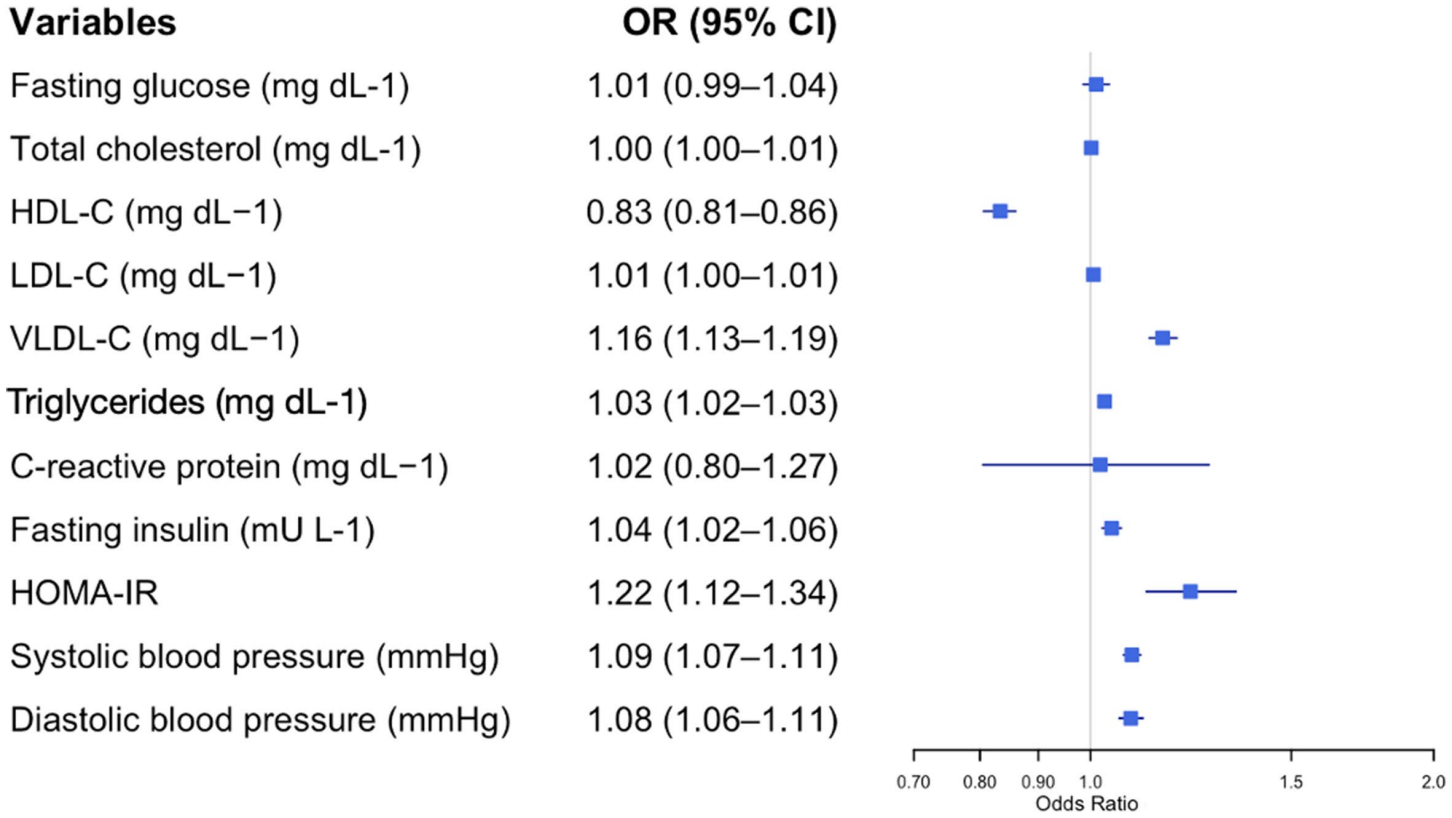
Forest plot of adjusted odds ratios (ORs) and 95% confidence intervals (CIs) for blood parameters and metabolic syndrome risk in adolescents with obesity. Each row displays a specific index, with the square dot representing the adjusted-for-age odds ratio and the horizontal line extending from the dot indicating the 95% confidence interval. The plot includes a vertical reference line at an OR of 1.0, representing no effect. Predictors with confidence intervals that do not cross this line suggest a statistically significant association with obesity risk. The numerical values of the odds ratio and their CI are placed next to each index. HDL-C, high-density lipoprotein cholesterol; LDL-C, low-density lipoprotein cholesterol; VLDL-C, very low-density lipoprotein cholesterol; HOMA-IR, Homeostasis Model Assessment Index-Insulin Resistance.

**Figure 3:**
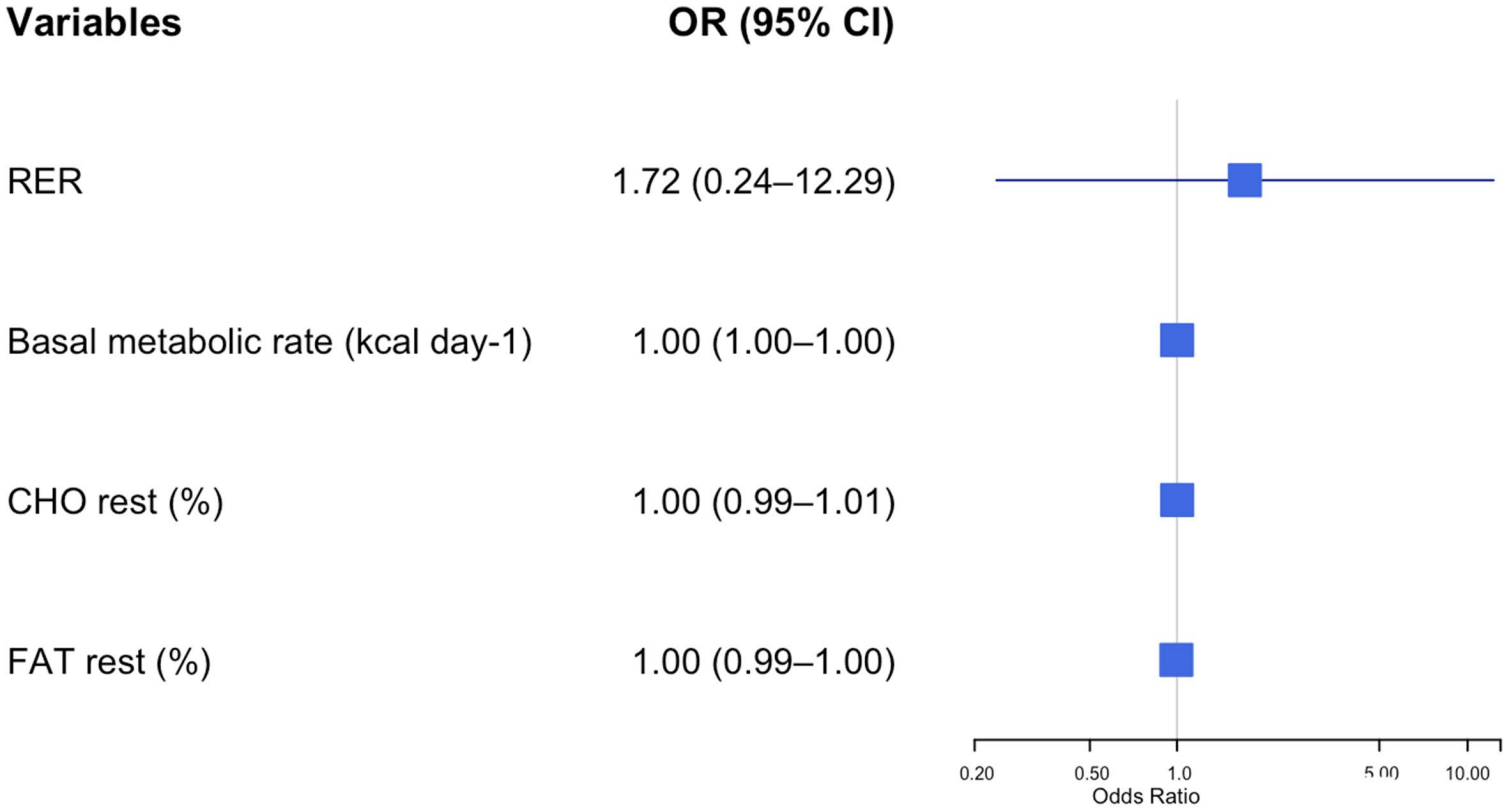
Forest plot of adjusted odds ratios (ORs) and 95% confidence intervals (CIs) for resting energy expenditure parameters and metabolic syndrome risk in adolescents with obesity. Each row displays a specific index, with the square dot representing the adjusted-for-age odds ratio and the horizontal line extending from the dot indicating the 95% confidence interval. The plot includes a vertical reference line at an OR of 1.0, representing no effect. Predictors with confidence intervals that do not cross this line suggest a statistically significant association with obesity risk. The numerical values of the odds ratio and their CI are placed next to each index. RER, respiratory exchange ratio; CHO, resting carbohydrate oxidation; FAT, resting fat oxidation.

**Figure 4:**
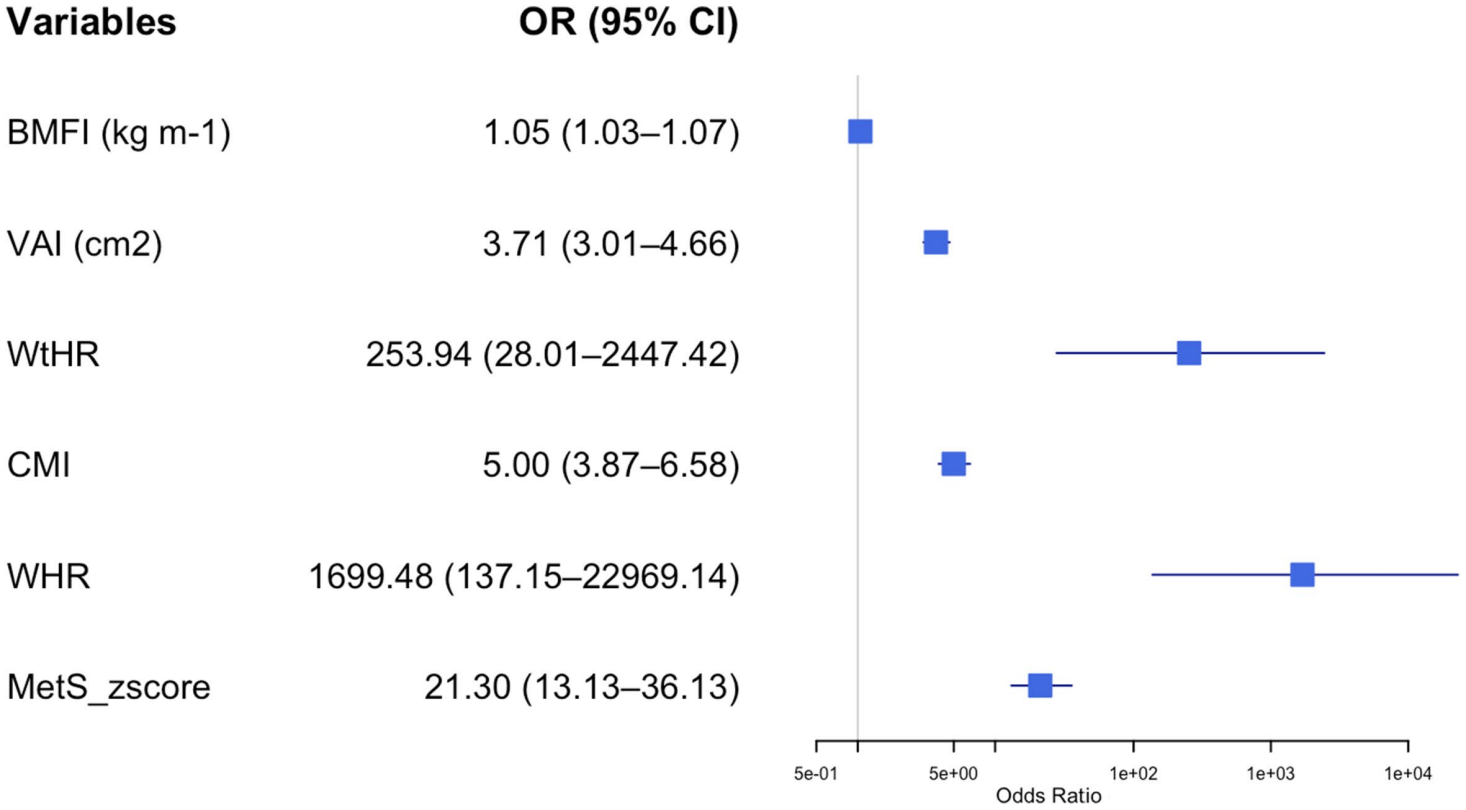
Forest plot of adjusted odds ratios (ORs) and 95% confidence intervals (CIs) for body composition parameters and metabolic syndrome risk in adolescents with obesity. Each row displays a specific index, with the square dot representing the adjusted-for-age odds ratio and the horizontal line extending from the dot indicating the 95% confidence interval. The plot includes a vertical reference line at an OR of 1.0, representing no effect. Predictors with confidence intervals that do not cross this line suggest a statistically significant association with obesity risk. The numerical values of the odds ratio and their CI are placed next to each index. BMFI, body mass fat index; VAI, visceral adiposity index; WHtR, waist-to-height ratio; CMI, cardiometabolic index; MetS_zscore, metabolic syndrome z score.

After adjusting for age and sex, logistic regression analysis identified several variables significantly associated with the presence of metabolic syndrome (MetS) (Figures 1–4).

Figure 1 illustrates the impact of various anthropometric and body composition parameters on the odds of having MetS in adolescents with obesity. All the parameters were directly related to the odds of having MetS. However, among the various parameters, higher BMI z-scores were strongly associated with increased odds of MetS (OR = 2.22, 95% CI: 1.61–3.09, *p* < 0.001). Conversely, higher FFM (%) was associated with reduced odds of MetS (OR = 0.96, 95% CI: 0.93–0.99, *p* = 0.003), indicating a protective role.

Figure 2 illustrates the impact of different cardiometabolic parameters on the odds of having MetS in adolescents with obesity. All the parameters, except for the HDL-C, were directly related to the odds of having MetS. A stronger association was found for the HOMA-IR (OR = 1.22, 95% CI: 1.12–1.34, *p* < 0.001). Conversely, HDL-C was associated with reduced odds of MetS (OR = 0.83, 95% CI: 0.81–0.86, *p* = 0.003).

Figure 3 shows how the different resting substrate oxidation data affected the odds of having MetS in the study group. Higher CHOrest values were associated with an increased risk of MetS (OR = 21.489, 95% CI: 2.46–190.5, *p* = 0.006).

Figure 4 illustrates the impact of different MetS indexes on the odds of having MetS in adolescents with obesity. All the indexes were directly related to the odds of having MetS. However, this association was stronger for the WHR (OR = 1699.48, 95% CI: 137.2–22969.1, *p* < 0.001) and WtHR (OR = 235.9, 95% CI: 28.01–2447.42, *p* < 0.001) than for the other indexes. Supplementary file 1 provides a summary table including all adjusted odds ratios (ORs), 95% confidence intervals (CIs), and Cohen’s d values for the associations between the evaluated parameters and metabolic syndrome risk in adolescents with obesity.

### MetS risk score

Among the ten variables considered for the score, seven were selected by the model with a selection frequency greater than 60%: WHR, FFM (kg), FAT (%), age, BMI, BMR, and sex (Table 3). The metabolic syndrome risk score was then constructed by multiplying the standardised variables by the normalised coefficients (Equation 1):


(1) $$\begin{array}{l} \text{Risk score}=20\times \frac{\mathrm{WHR}-0.95}{0.08}+10\times \frac{\mathrm{FFM}\;(\mathrm{kg})-47.62}{9.88}\\ -12\times \frac{\mathrm{FAT}\;(\%)-65.8}{23.2}+5\times \frac{\mathrm{age}\;(y)-14.63}{2.07}+5\\ \times \frac{\mathrm{BMI}-37.92}{6.24}+6\times \frac{\mathrm{BMR}-1928}{363.5}+11\times (1\;\text{if female},0\;\text{if male}) \end{array}$$

**Table 3:** Variables selected for the novel metabolic syndrome index.

Variables	Selection frequency	Mean coefficient	Normalized coefficient
WHR	100%	0.54	20
FFM (kg)	93%	0.26	10
FAT (%)	86%	−0.31	−12
Age	79%	0.13	5
BMI (kg m^−2^)	77%	0.14	5
BMR (kcal day^−1^)	72%	0.16	6
Female sex	65%	0.29	11

Each row represented the variables selected from the model for the novel index. The table shows the selection frequency (percentage of times selected across 100 resampled models) for each variable, the mean estimated coefficient, and the normalised coefficient. WHR, waist-to-hip ratio; FAT, resting fat oxidation; BMR, basal metabolic rate; FFM, fat-free mass; FM, fat mass; BMI, body mass index.

**Figure 5:**
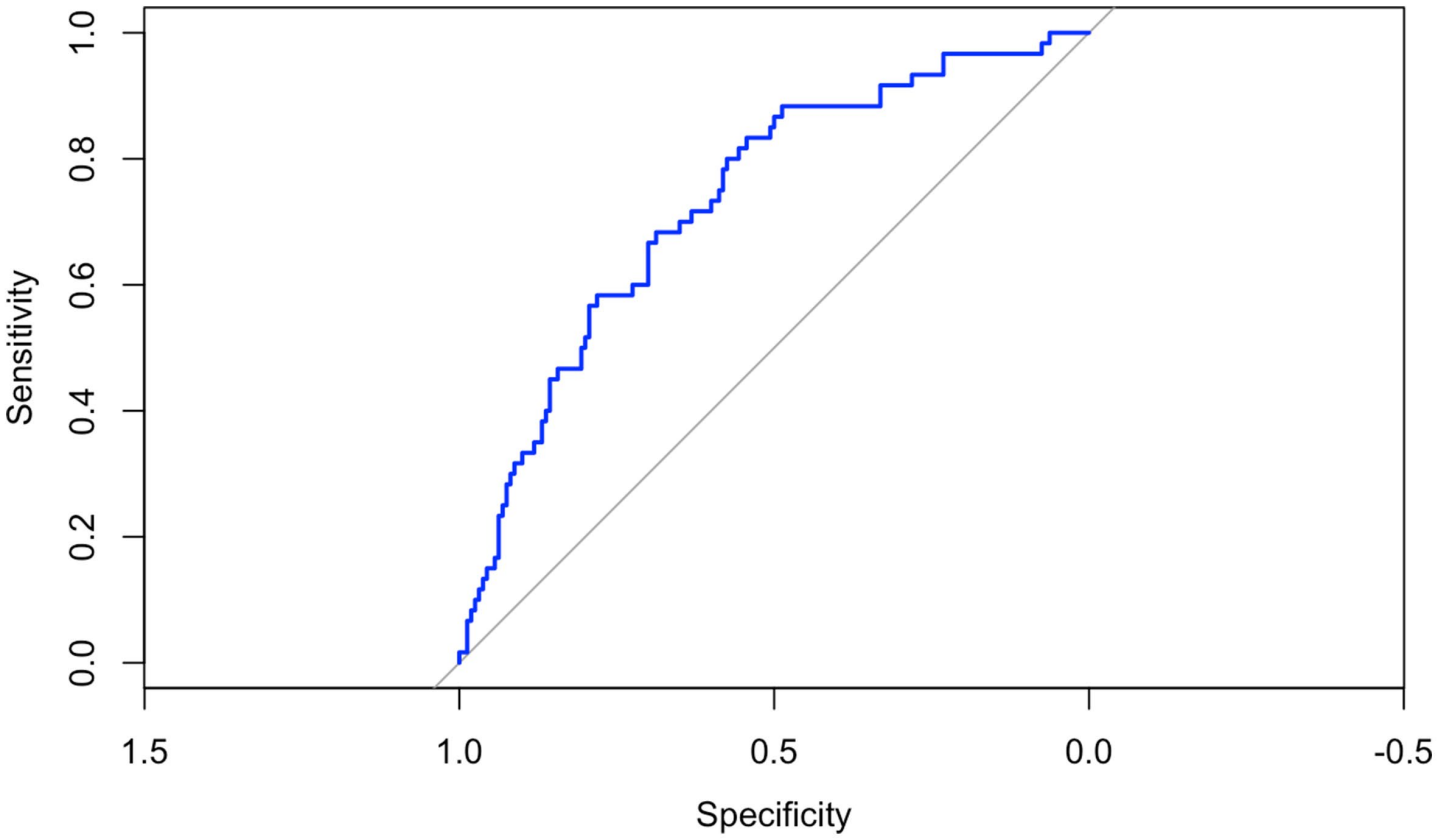
The receiver operating characteristic (ROC) curve of the novel index in predicting metabolic syndrome in adolescents with obesity in the testing set. The ROC curve illustrates the trade-off between sensitivity (true positive rate) and 1-specificity (false positive rate) across different cut-offs. The model is based on a risk score created by the LASSO logistic regression trained on 70% of the dataset and tested on the remaining 30% of participants. The area under the curve (AUC) was 0.73 (95% CI: 0.66–0.81), indicating a good discriminatory ability of the index in differentiating between adolescents with and without metabolic syndrome.

The resulting score ranged from −70.35 to 92.14 in the training dataset, with a mean of 6.74 (SD: 29.6). The optimal threshold for identifying individuals at risk of metabolic syndrome corresponded to a score of 1.85. Model performance was evaluated on the testing set, yielding an AUC of 0.73 (95% CI: 0.66–0.81), indicating a good discriminative ability (Figure 5). At the optimal cut-off, the model showed a sensitivity of 0.83 (95% CI: 0.71–0.92), a specificity of 0.54 (95% CI: 0.46–0.62), a positive predictive value (PPV) of 0.41 (95% CI: 0.32–0.50), and a negative predictive value (NPV) of 0.90 (95% CI: 0.82–0.95).

## Discussion

The main findings of the present study were: (i) adolescents with MetS exhibited higher values of WC and HC, as well as elevated BMI, FM, triglycerides, fasting insulin, and HOMA-IR compared to their peers without MetS; (ii) FFM (%) and high-density lipoprotein cholesterol (HDL-C) were protective factors for MetS; (iii) higher BMI z-scores, HOMA-IR, and resting CHO were identified as significant risk factors for the development of MetS. Moreover, this paper proposed a novel predictive index for MetS that incorporates direct measures of body composition and resting substrate oxidation, which demonstrated good discriminative ability in identifying the presence of MetS.

Our findings revealed that 202 participants (28%) in our sample met the diagnostic criteria for MetS. This prevalence aligns with previous cross-sectional studies in paediatric populations with obesity, where rates of MetS range from 10 to 38% (4, 5). WC was significantly higher in adolescents with MetS, reinforcing its role as an indirect marker of visceral adipose tissue (VAT) accumulation (20). Consistent with prior research (39, 44, 45), adolescents with MetS exhibited a higher prevalence of metabolic abnormalities, including elevated triglycerides, hyperinsulinemia, insulin resistance, and reduced levels of HDL-C. These data suggest that increased central adiposity, reflected by greater WC, may contribute to the development of insulin resistance and hyperinsulinemia through several well-established mechanisms (46). VAT is highly metabolically active and exhibits increased lipolytic activity, leading to elevated circulating free fatty acids (47). Free fatty acids, in turn, impair insulin-mediated glucose uptake in peripheral tissues partially by the secretion of pro-inflammatory cytokines, which interfere with insulin signalling pathways (47, 48). All together, these alterations contribute to impaired insulin action and may promote early metabolic dysfunction, although this mechanistic pathway was not directly assessed in the present study.

The second key finding of our study was the identification of several significant risk and protective factors associated with the development of MetS in a large cohort of adolescents with obesity. Notably, our analysis highlighted two main protective factors. The first is FFM (%). FFM plays a crucial protective role in the development of MetS in adolescents with obesity. Indeed, higher FFM is associated with improved insulin sensitivity, enhanced glucose uptake, and increased resting energy expenditure, all of which contribute to a more favourable cardiometabolic profile. This is partially supported by our findings, which revealed significant differences in body composition parameters between adolescents with and without MetS. Moreover, skeletal muscle, the main component of FFM, serves as a primary site for glucose disposal and lipid oxidation, playing a central role in maintaining metabolic homeostasis (49, 50). Regular physical activity, particularly resistance and aerobic training, promotes muscle hypertrophy and mitochondrial adaptations, thereby increasing FFM (10, 51). These physiological changes enhance substrate utilisation efficiency and may substantially lower the risk of developing MetS later in life (52). The second protective factor identified is HDL-C. Higher levels of HDL-C were associated with a significantly reduced risk of MetS (OR < 1), underscoring the well-established protective role of HDL in metabolic health. Beyond its role in reverse cholesterol transport, HDL also exerts anti-inflammatory and antioxidant effects that may enhance insulin sensitivity and lower cardiometabolic risk (53). These findings underscore the importance of comprehensive metabolic profiling in adolescents with obesity to detect early metabolic alterations. Identifying both risk and protective factors can inform personalised prevention strategies aimed at improving long-term metabolic health and reducing the progression of MetS into adulthood. Among the most relevant risk factors, a higher rate of resting CHO emerged as a novel and independent predictor of MetS. This observation suggests that adolescents with obesity who predominantly rely on carbohydrates as an energy source at rest may display impaired metabolic flexibility, potentially indicating early alterations in mitochondrial efficiency or insulin signalling (10, 14). From a physiological perspective, elevated resting CHO oxidation may reflect a reduced capacity for lipid oxidation, possibly linked to diminished mitochondrial oxidative function or a blunted response to insulin (10, 14). Such metabolic shifts may favour glycolytic pathways even in the absence of acute energy demand. Moreover, several studies showed that resting metabolic inflexibility and elevated resting CHO are not features of obesity per se, but rather distinctive traits of youth with metabolically unhealthy obesity (13, 54). As previously suggested, these individuals also exhibit poorer insulin sensitivity compared to their peers with metabolically healthy obesity, findings that are partially supported by our results. In our adolescent sample, those with MetS displayed a worse glycaemic profile than those without MetS, despite both groups being affected by obesity. Nonetheless, several confounding factors may influence substrate utilisation at rest, including habitual diet, recent food intake, cardiorespiratory fitness, and physical activity levels (17, 55). These variables can affect insulin sensitivity and substrate availability, thereby modulating the balance between fat and carbohydrate oxidation. While some of these aspects were not directly accounted for in the present analysis, they warrant consideration in future investigations to clarify the underlying mechanisms and strengthen the interpretability of resting CHO oxidation as a marker of cardiometabolic risk. However, the strength of our model lies in having adjusted the odds ratio for age, sex, and the presence of MetS.

However, current findings in paediatric populations remain inconsistent, partly due to the limited consideration of confounding factors such as cardiorespiratory fitness and substrate utilisation during aerobic exercise (56). Elevated BMI z-scores and higher values of the HOMA-IR were also significantly associated with the presence of MetS, in line with previous literature emphasising the central role of excess adiposity and insulin resistance in the pathogenesis of metabolic dysfunction (57, 58). The ORs for these variables indicated a markedly increased likelihood of developing MetS, reinforcing their relevance in early clinical risk stratification.

The last important finding of our study was the development of a novel predictive index for MetS, which integrates WHR, FFM, resting FAT (%), age, BMI, BMR, and sex. This composite index yielded an AUC of 0.73, indicating moderate to good discriminative ability for identifying individuals at risk of MetS. The inclusion of BMR, FFM, and resting FAT (%) represents a key innovation of this model, as these parameters reflect essential aspects of metabolic function that are typically overlooked in standard clinical assessments. Both BMR and FFM are major determinants of total energy expenditure (17) while resting FAT (%) is closely related to metabolic flexibility (12). Moreover, reduced resting FAT (%) has been associated with an increased risk of developing metabolic dysregulation and insulin resistance later in life (13). However, in our cohort, we did not observe significant differences in resting FAT (%) between adolescents with and without MetS. In comparison, commonly used indexes such as BMFI, VAI, WtHR, CMI, and the MetS z-score have demonstrated AUC values ranging from 0.55 to 0.77 in paediatric populations (39, 45). While these indexes provide practical screening tools, they do not account for metabolic and bioenergetic resting parameters. In contrast, our model incorporates both structural components (e.g., FFM, BMI, WHR) and resting energetics parameters (e.g., BMR, resting FAT oxidation), offering a more integrated approach to metabolic risk assessment. Although the predictive performance of our index is comparable to that of existing models, its inclusion of physiologically relevant variables may enhance early risk stratification when used in conjunction with traditional markers. Further validation in larger and more diverse cohorts is warranted to optimise its predictive value and explore its clinical applicability in preventive care. Notably, while the model demonstrated good sensitivity, its specificity was low, suggesting that further investigation and external validation are necessary to improve its ability to accurately exclude individuals without metabolic syndrome and enhance its overall clinical performance. Moreover, external replication studies are necessary to confirm the model’s stability, particularly to investigate the wide confidence intervals observed for some predictors (55).

Our study has several limitations that should be acknowledged. First, longitudinal research is necessary to evaluate the ability of our index to predict the progression of MetS over time in adolescents with obesity. Second, we did not employ dual-energy X-ray absorptiometry (DEXA), the gold standard for body composition analysis, due to the large sample size, cost constraints, and concerns related to radiation exposure in paediatric populations. Instead, we used WC as a surrogate marker of central obesity. Although WC is widely adopted in clinical research, it may be subject to measurement variability. To mitigate this, all anthropometric assessments were conducted by trained and experienced healthcare professionals, enhancing data consistency and reliability.

Moreover, although BIA is less accurate than DEXA (which represents the gold standard), it offers a feasible, non-invasive, and scalable method for estimating body composition, particularly in large cohorts and standard clinical settings. Its affordability and ease of use make it especially valuable in paediatric obesity management, where access to advanced imaging modalities is often constrained. Similarly, although indirect calorimetry is not the gold standard for assessing substrate oxidation, it remains a validated and widely adopted method. Together, these tools offer a pragmatic yet scientifically sound approach, striking an important balance between methodological rigour and practical applicability. Emphasising this balance strengthens the translational relevance of the study, supporting its implementation in real-world settings where resources and time are often limited.

In conclusion, our study identified FFM and HDL-C as significant protective factors against the development of MetS in adolescents with obesity. In contrast, increased WC, BMI, HOMA-IR, and resting RER emerged as key risk factors. These findings emphasise the critical role of body composition, both as a protective and risk-related component, in the pathogenesis of MetS. Moreover, they underscore the importance of promoting regular aerobic and resistance exercise in this population as a targeted strategy to modify risk factors and reduce the likelihood of developing MetS. Importantly, we propose a novel predictive index that incorporates WHR, FFM, resting FAT (%), BMI, BMR, and sex, which demonstrates good discriminatory power and provides a more comprehensive assessment of metabolic risk compared to traditional anthropometric-based indexes. Future longitudinal studies are, however, necessary to understand how changes in this novel predictive index over time might be usefully employed in guiding personalised therapeutic interventions for this clinical condition in a more effective manner.

## Data Availability

The datasets analyzed in this study cannot be made publicly available as they include sensitive information, but they can be made available upon reasonable request of interested researchers to the corresponding author, who will forward a data transfer agreement request to the relevant Ethical Committee. Requests can be addressed to Dr. Alessandro Sartorio (sartorio@auxologico.it).
