# Supplementary material for: The role of body composition, cardiometabolic parameters, and resting substrate oxidation in protecting against metabolic syndrome in adolescents with obesity
Source: Front Nutr. 2025 Jul 30;12:1624696. doi: 10.3389/fnut.2025.1624696 (PMC12345374; doi:10.3389/fnut.2025.1624696)
Supplement: Supplementary file 1 [file Table_1.docx]

**Supplementary Table 1.** Adjusted odds ratios (ORs), 95% confidence intervals (CIs) and Cohen’s d for parameters and metabolic syndrome risk in adolescents with obesity.

| Variable | OR | IC 95% (Lower) | IC 95% (Upper) | P-value | Cohen's d |
| --- | --- | --- | --- | --- | --- |
| Body weight (kg) | 1.02 | 1.01 | 1.03 | **<0.001** | 0.013 |
| BMI | 1.06 | 1.03 | 1.09 | **<0.001** | 0.033 |
| BMI z-score | 2.22 | 1.61 | 3.09 | **<0.001** | 0.440 |
| FFM (kg) | 1.05 | 1.02 | 1.07 | **<0.001** | 0.025 |
| FFM (%) | 0.96 | 0.93 | 0.99 | **0.003** | 0.024 |
| FM (kg) | 1.03 | 1.02 | 1.04 | **<0.001** | 0.015 |
| FM (%) | 1.04 | 1.01 | 1.08 | **0.003** | 0.024 |
| Basal metabolic rate | 1.01 | 0.98 | 1.04 | 0.658 | 0.004 |
| Fasting glucose | 1.01 | 0.99 | 1.04 | 0.388 | 0.006 |
| Total cholesterol | 1.00 | 1.00 | 1.01 | 0.600 | 0.001 |
| HDL-C | 0.83 | 0.81 | 0.86 | **<0.001** | 0.100 |
| LDL-C | 1.01 | 1.00 | 1.01 | **0.041** | 0.003 |
| VLDL-C | 1.16 | 1.13 | 1.19 | **<0.001** | 0.080 |
| Triglycerides | 1.03 | 1.02 | 1.03 | **<0.001** | 0.016 |
| C-reactive protein | 1.02 | 0.80 | 1.27 | 0.862 | 0.011 |
| Fasting insulin | 1.04 | 1.02 | 1.06 | **<0.001** | 0.024 |
| HOMA-IR | 1.22 | 1.12 | 1.34 | **<0.001** | 0.111 |
| Systolic blood pressure | 1.09 | 1.07 | 1.11 | **<0.001** | 0.046 |
| Diastolic blood pressure | 1.09 | 1.06 | 1.11 | **<0.001** | 0.045 |
| RER | 1.72 | 0.24 | 12.29 | 0.591 | 0.298 |
| Basal metabolic rate | 1.001 | 1.001 | 1.002 | **<0.001** | 0.001 |
| CHO rest (%) | 1.00 | 0.99 | 1.00 | 0.670 | 0.001 |
| FAT rest (%) | 1.00 | 0.99 | 1.00 | 0.197 | 0.003 |
| BMFI | 1.05 | 1.03 | 1.07 | **<0.001** | 0.024 |
| VAI | 3.71 | 3.01 | 4.66 | **<0.001** | 0.723 |
| WtHR | 253.94 | 28.01 | 2447.42 | **<0.001** | 3.053 |
| CMI | 5.00 | 3.87 | 6.58 | **<0.001** | 0.887 |
| WHR | 1699.48 | 137.15 | 22969.14 | **<0.001** | 4.101 |
| MetS z-score | 21.30 | 13.13 | 36.13 | **<0.001** | 1.686 |

*BMI, body mass index; FFM, fat-free mass; FM, fat mass; HDL-C, high-density lipoprotein cholesterol; LDL-C, low-density lipoprotein cholesterol; VLDL-C, very low-density lipoprotein cholesterol; HOMA-IR, Homeostasis Model Assessment Index-Insulin Resistance; RER, resting exchange ratio; CHOrest, carbohydrate oxidation at rest; FATrest, fat oxidation at rest; BMFI, body mass fat index; VAI, visceral adiposity index; WHtR, waist-to-height ratio; CMI, cardiometabolic index; WHR, waist-to-hip ratio; MetS_zscore, metabolic syndrome z score.*
